# Diagnostic accuracy of 3 urine lipoarabinomannan tuberculosis assays in HIV-negative outpatients

**DOI:** 10.1172/JCI140461

**Published:** 2020-09-28

**Authors:** Tobias Broger, Mark P. Nicol, George B. Sigal, Eduardo Gotuzzo, Alexandra J. Zimmer, Shireen Surtie, Tatiana Caceres-Nakiche, Anna Mantsoki, Elena Ivanova Reipold, Rita Székely, Michael Tsionsky, Judith van Heerden, Tatiana Plisova, Kinuyo Chikamatsu, Todd L. Lowary, Abraham Pinter, Satoshi Mitarai, Emmanuel Moreau, Samuel G. Schumacher, Claudia M. Denkinger

**Affiliations:** 1 FIND, Geneva, Switzerland.; 2 Division of Tropical Medicine, Center for Infectious Diseases, Heidelberg University Hospital, Heidelberg, Germany.; 3 Division of Infection and Immunity, School of Biomedical Sciences, University of Western Australia, Perth, Australia.; 4 Division of Medical Microbiology, University of Cape Town, Cape Town, South Africa.; 5 Meso Scale Diagnostics LLC, Rockville, Maryland, USA.; 6 Cayetano Heredia University, Lima, Peru.; 7 Department of Mycobacterium Reference and Research, Research Institute of Tuberculosis, Japan Anti-Tuberculosis Association, Tokyo, Japan.; 8 Department of Chemistry, University of Alberta, Edmonton, Alberta, Canada.; 9 Institute of Biological Chemistry, Academia Sinica, Taipei, Taiwan.; 10 Public Health Research Institute Center, New Jersey Medical School, Rutgers University, Newark, New Jersey, USA.

**Keywords:** Infectious disease, Antigen, Diagnostics, Tuberculosis

## Abstract

**BACKGROUND:**

Inadequate tuberculosis (TB) diagnostics are a major hurdle in the reduction of disease burden, and accurate point-of-care tests (POCTs) are urgently needed. We assessed the diagnostic accuracy of Fujifilm SILVAMP TB lipoarabinomannan (FujiLAM) POCT for TB diagnosis in HIV-negative outpatients and compared it with Alere Determine TB LAM Ag (AlereLAM) POCT and a laboratory-based ultrasensitive electrochemiluminescence LAM research assay (EclLAM).

**METHODS:**

In this multicenter diagnostic test accuracy study, we recruited HIV-negative adults with symptoms suggestive of pulmonary TB presenting to outpatient health care centers in Peru and South Africa. Urine samples were tested using FujiLAM, AlereLAM, and EclLAM, and the diagnostic accuracy was assessed against a microbiological reference standard (MRS) and a composite reference standard.

**RESULTS:**

Three hundred seventy-two HIV-negative participants were included and the prevalence of microbiologically confirmed TB was 30%. Compared with the MRS, the sensitivities of AlereLAM, FujiLAM, and EclLAM were 10.8% (95% confidence interval [CI] 6.3%–18.0%), 53.2% (95% CI 43.9%–62.1%), and 66.7% (95% CI 57.5%–74.7%), respectively. The specificities of AlereLAM, FujiLAM, and EclLAM were 92.3% (95% CI 88.5%–95.0%), 98.9% (95% CI 96.7%–99.6%), and 98.1% (95% CI 95.6%–99.2%), respectively. Positive likelihood ratios of AlereLAM, FujiLAM, and EclLAM were 1.4, 46.2, and 34.8, respectively, and positive predictive values were 37.5%, 95.2%, and 93.7%, respectively.

**CONCLUSION:**

Compared with AlereLAM, FujiLAM detected 5 times more patients with TB in HIV-negative participants, had a high positive predictive value, and has the potential to improve rapid diagnosis of TB at the point-of-care. EclLAM demonstrated that additional sensitivity gains are possible, which highlights LAM’s potential as a biomarker. Additional research is required to assess FujiLAM’s performance in prospective cohorts, its cost-effectiveness, and its impact in real-world clinical settings.

**FUNDING:**

Global Health Innovative Technology Fund, the UK Department for International Development, the Dutch Ministry of Foreign Affairs, the Bill and Melinda Gates Foundation, the Australian Department of Foreign Affairs and Trade, the German Federal Ministry of Education and Research through Kreditanstalt für Wiederaufbau, and the NIH and National Institute of Allergy and Infectious Diseases.

## Graphical abstract

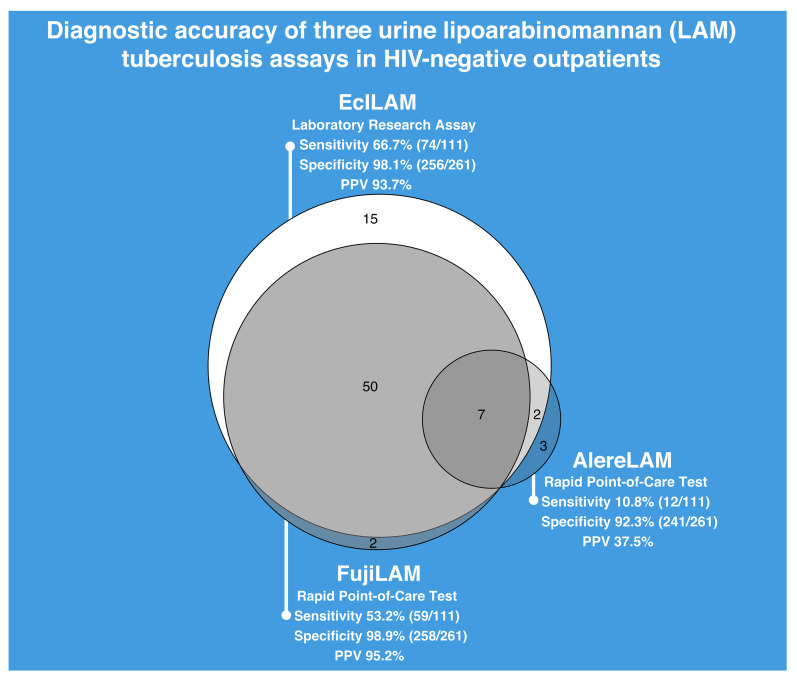

## Introduction

Tuberculosis (TB) is the leading single infectious cause of death worldwide, with more than 1.5 million deaths in 2018 (1). The high rate of unreported TB (estimated at 3.0 million cases) indicates that inadequate diagnostics are a major hurdle in the reduction of disease burden (1). To address this gap, the World Health Organization (WHO) put forth a set of target product profiles (TPPs) (2, 3) to encourage the development of point-of-care tools to enhance TB case detection. One such TPP is a non–sputum biomarker test for the purpose of initiating TB treatment during the same clinical encounter (2). An interesting biomarker for this application is the lipoarabinomannan (LAM) antigen found in mycobacterial cell walls (4, 5).

The Alere Determine TB LAM antigen assay (AlereLAM) is a TB point-of-care test (POCT) that detects LAM in urine using a simple disposable lateral flow assay. Currently, AlereLAM is the only instrument-free POCT recommended by the WHO for TB. However, due to its limited sensitivity, its recommended use is limited to assisting in the diagnosis of active TB in people living with HIV in advanced stages (6–8). Despite the limited sensitivity, AlereLAM-guided initiation of anti-TB treatment reduced mortality in immunocompromised, hospitalized people living with HIV (9, 10). AlereLAM is not recommended for diagnosis of TB in people living with HIV with CD4 greater than 200 cells/μL due to a suboptimal sensitivity of 16% in this population (6, 7). Performance in HIV-negative patients is very poor, with reported estimated sensitivities ranging from 4% to 31% (11–15).

Fujifilm recently developed a next-generation POCT, the Fujifilm SILVAMP TB LAM test (FujiLAM). To improve sensitivity while maintaining high specificity, FujiLAM uses a pair of high-affinity monoclonal antibodies selected to detect LAM presenting the *Mycobacterium tuberculosis*–specific (*Mtb*-specific) 5-Methylthio-D-xylofuranose epitope (MTX-LAM), and employs a silver-amplification step (16–18). A recent meta-analysis of 1595 HIV-positive inpatients and outpatients confirmed FujiLAM’s superiority, demonstrating a sensitivity of 71%, twice that of AlereLAM (19). Further, FujiLAM showed good sensitivity for the detection of extrapulmonary TB (EPTB) ranging from 47% to 94% across different forms of ETB (20) and could have rapidly diagnosed TB in up to 89% of HIV-positive inpatients who died within 12 weeks (21).

A non–sputum-based biomarker test would also benefit HIV-negative patients, particularly those with extrapulmonary TB or those unable to produce sputum. This study aimed to assesses FujiLAM’s performance in HIV-negative adults with presumptive pulmonary TB. To better understand the relationship between analytical detection limits and clinical sensitivity, the results from FujiLAM were compared with the results from a research assay (EclLAM) employing the same antibodies, but using a more sensitive laboratory immunoassay platform employing electrochemiluminescence (ECL) (14).

## Results

**Figure 1:**
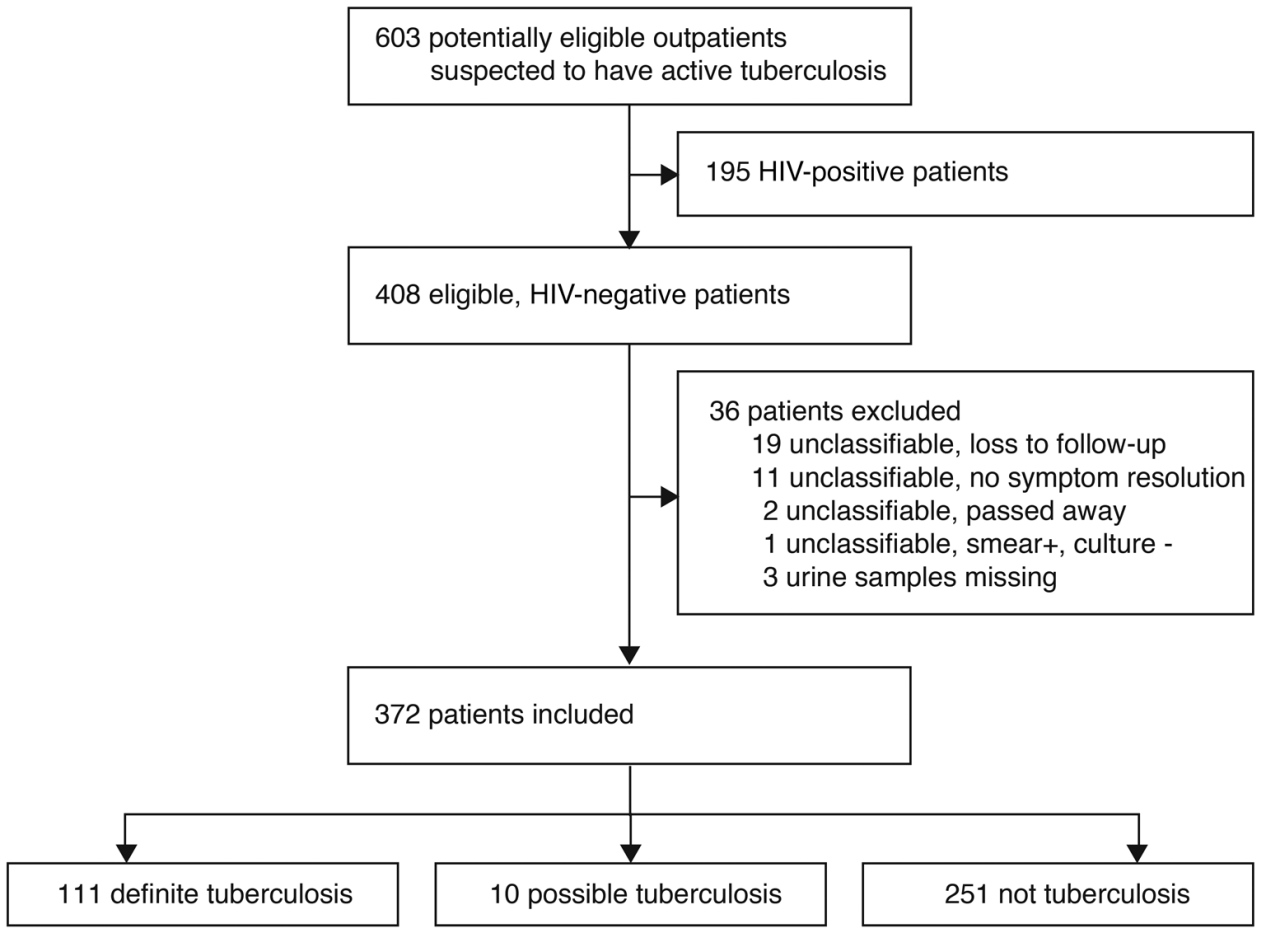
Study flow diagram.

Between February 9, 2017, and October 4, 2017, 603 potentially eligible participants were screened. A total of 408 HIV-negative participants met inclusion criteria and 372 were included in the analyses (Figure 1). Of these, 30% (111/372) were classified as definite TB, 3% (10/372) as possible TB, and 67% (251/372) as not TB (Table 1). Prevalence of definite TB was higher in Peru (43%) compared with South Africa (17%). Most participants were young adults (median age 32 years) and 14% had a history of prior TB disease. In participants with definite TB, 68% (76/111) had at least one positive fluorescence sputum smear microscopy (SSM) result. Peruvian participants with TB had shorter mycobacterial growth indicator tube (MGIT) liquid culture time to detection and a larger proportion of patients had positive SSM and Xpert results compared with patients from South Africa, suggesting higher mycobacterial load in sputum (Table 1). None of the patients with definite TB had a positive blood culture and only 4% (4/111) had a positive urine Xpert result, with all of the latter also having positive sputum Xpert and culture results.

**Figure 2:**
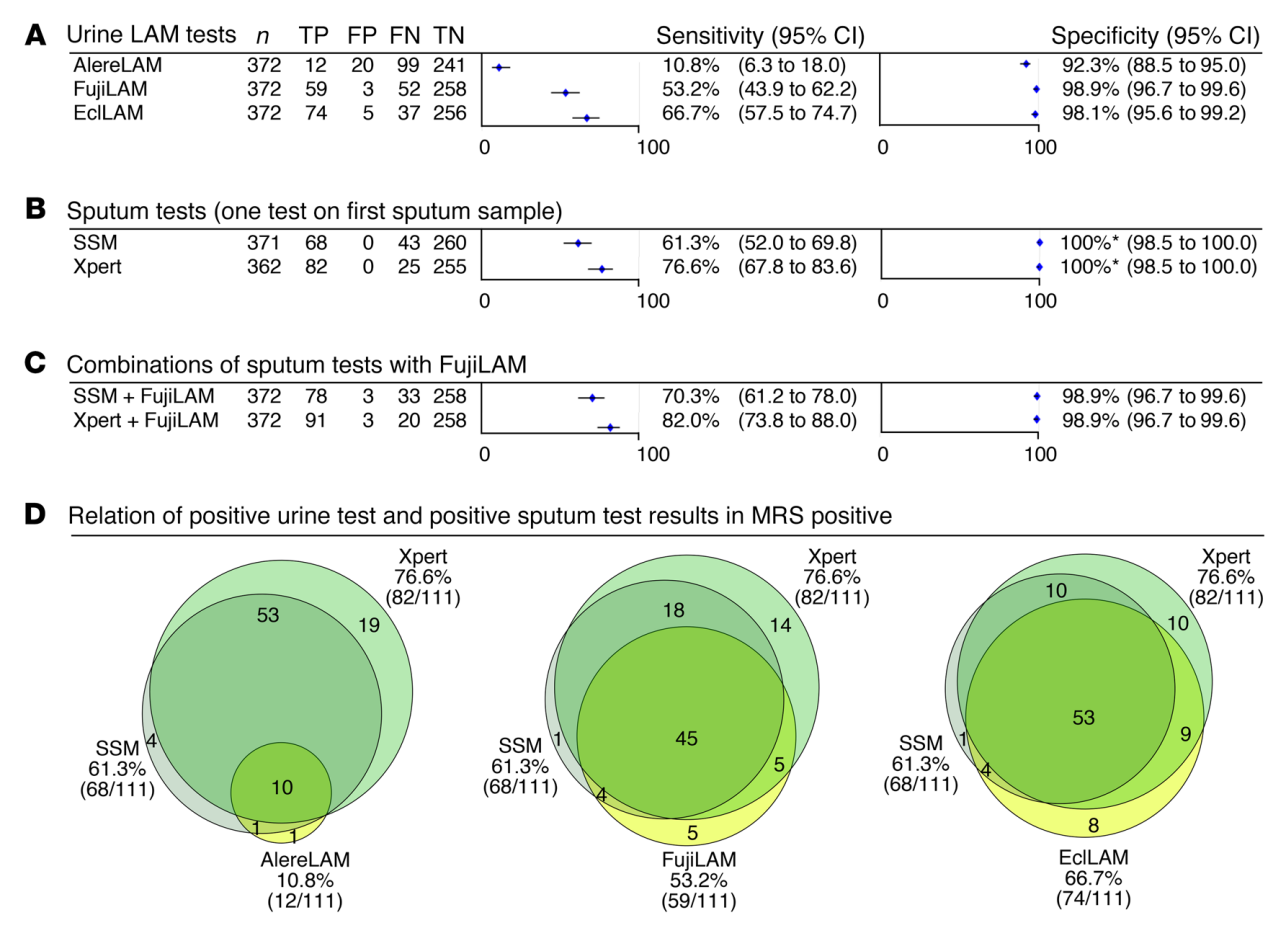
Diagnostic accuracy against microbiological reference standards. (**A**) Urine LAM tests, (**B**) sputum tests, (**C**) combinations of sputum tests with FujiLAM, and (**D**) positive urine LAM tests in relation to positive sputum tests. *SSM and Xpert were part of the microbiological reference standard and therefore specificity is 100%. TP, true positive; FP, false positive; FN, false negative; TN, true negative; AlereLAM, Alere Determine TB LAM Ag assay; FujiLAM, Fujifilm SILVAMP TB LAM assay; EclLAM, electrochemiluminescence-based LAM detection assay; SSM, sputum smear microscopy; Xpert, GeneXpert MTB/RIF assay; MRS, microbiological reference standard.

The index test failure rate number was very low (1 repeat FujiLAM and no repeats for AlereLAM). Results of the diagnostic accuracy of urine LAM-based assays (AlereLAM, FujiLAM and EclLAM), sputum-based assays (Xpert and SSM), and combinations of these assays are shown in Figure 2 and in Supplemental Figure 3 (supplemental material available online with this article; https://doi.org/10.1172/JCI140461DS1). Overall, compared with the microbiological reference standard (MRS), the sensitivities of AlereLAM, FujiLAM, and EclLAM were 10.8% (12/111; 95% confidence interval [CI] 6.3%–18.0%), 53.2% (59/111; 95% CI 43.9%–62.2%), and 66.7% (74/111; 95% CI 57.5%–74.7%), respectively (Figure 1). The sensitivity of urine FujiLAM and sputum Xpert in combination was 82.0% (91/111; 95% CI 73.8%–88.0%) and the sensitivity of urine FujiLAM in combination with a single SSM was 70.3% (78/111; 95% CI 61.2%–78.0%) and higher than Xpert alone (Figure 2, B and C). Using the composite reference standard (CRS), the sensitivities of all assays were not substantially changed (FujiLAM 48.8%, EclLAM 62.0%, AlereLAM 12.4%) (Supplemental Table 3).

All tests, except AlereLAM, reached specificities of 98% or higher in the MRS-based analysis (AlereLAM 92.3% (241/261; 95% CI 88.5%–95.0%), FujiLAM 98.9% (258/261; 95% CI 96.7%–99.6%), EclLAM 98.1% (256/261; 95% CI 95.6%–99.2%). When comparing results from the CRS-based analysis to the MRS-based analysis, specificity remained largely unchanged for all assays (AlereLAM 93.2 [234/251], FujiLAM 98.8% [248/251], EclLAM 98.4% [247/251]) (Supplemental Table 3). Against the MRS at study prevalence of 30%, the positive predictive values (PPVs) of AlereLAM, FujiLAM, and EclLAM were 37.5%, 95.2%, and 93.7%, respectively. When assuming a lower pretest probability of 20%, the PPV of AlereLAM, FujiLAM and EclLAM were 26.1%, 92.0%, and 89.7% respectively (Table 2). Positive likelihood ratios (LR+) were 46.2 for FujiLAM and 1.4 for AlereLAM. When assuming 20% pretest probability, the negative predictive values (NPVs) of AlereLAM, FujiLAM, and EclLAM were 80.5%, 89.4%, and 92.2%, respectively (Table 2). Negative likelihood ratios (LR−) were 0.47 for FujiLAM and 0.97 for AlereLAM. Fagan nomograms illustrating pretest and posttest probabilities are available in Supplemental Figure 4.

**Figure 3:**
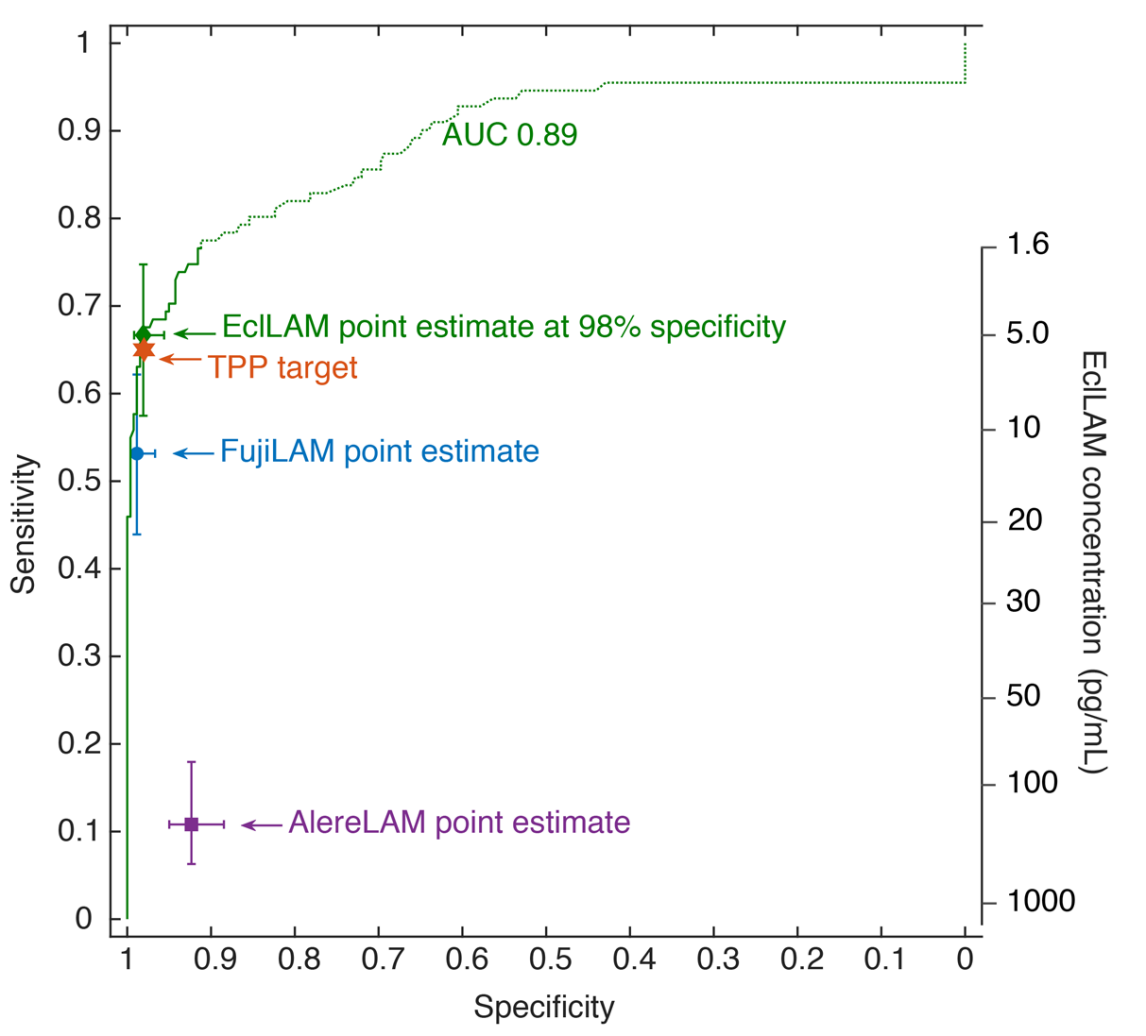
ROC analysis of the EclLAM concentration data compared with FujiLAM, AlereLAM, and EclLAM performance. The EclLAM concentration for the ROC curve is indicated on the secondary *y* axis. The ROC curve was restricted by the LOD (1.6 pg/mL) of the EclLAM assay, meaning that lower concentrations could not be measured so that the upper part of the ROC curve (green dotted line) should be treated with caution. *n* = 372 patients. TPP, target product profile.

Figure 3 shows the receiver operating characteristic (ROC) curve of the quantitative EclLAM assay and highlights the point estimates of AlereLAM, FujiLAM, and EclLAM (at a cutoff of 5.2 pg/mL) in comparison with the TPP performance target. Using the conversion scale based on the EclLAM calibration curve (Supplemental Figure 2) in Figure 3, we estimate that the LAM threshold of FujiLAM is 10–20 pg/mL and at least 10 times below the threshold of AlereLAM. Data suggest that a threshold around 5 pg/mL or below is required to meet the TPP sensitivity target.

**Figure 4:**
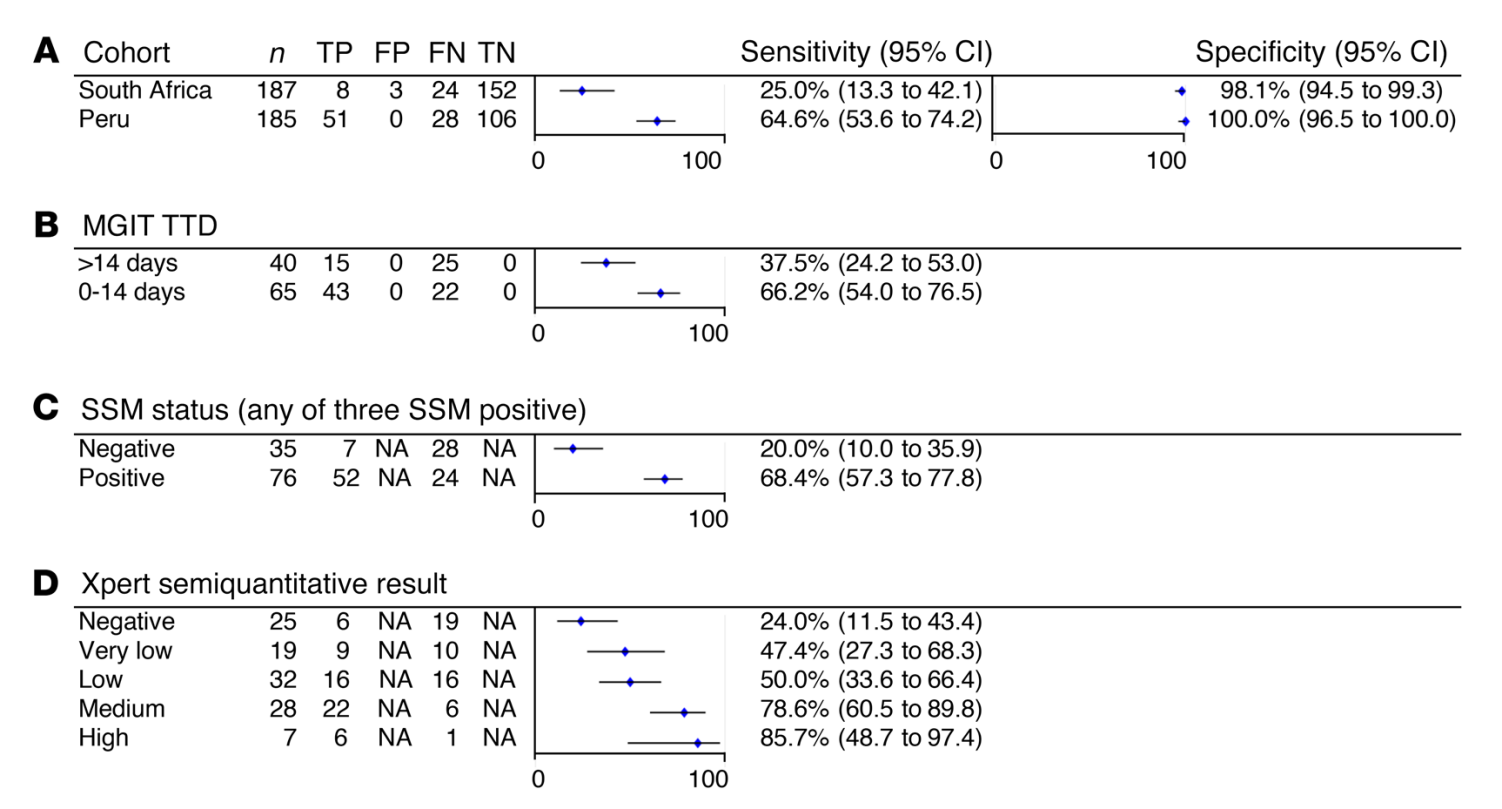
Subgroup analysis of FujiLAM. Sensitivity and specificity against microbiological reference standard of (**A**) FujiLAM by study site, (**B**) FujiLAM by MGIT TTD, (**C**) FujiLAM by SSM status, and (**D**) FujiLAM by semiquantitative Xpert result.

Subgroup analysis revealed that FujiLAM sensitivity was higher in Peru (64.6%) compared with South Africa (25.0%), and this trend was also observed for AlereLAM and EclLAM (Figure 4A and Supplemental Figures 5 and 6). Subgroup analyses per MGIT TTD (Figure 4B), SSM status (Figure 4C), and semiquantitative GeneXpert MTB/RIF (Xpert) results (Figure 4D) indicate that FujiLAM sensitivity increases as mycobacterial load in sputum increases, and again this trend was confirmed with AlereLAM and EclLAM (Supplemental Figures 5 and 6).

Importantly, FujiLAM fails to detect 31.6% (24/76) of SSM-positive patients when using 3 SSM results as the basis (Figure 4C) or 27.9% (19/68) when using 1 SSM result as the basis (Figure 2D). On the other hand, FujiLAM detected 23.3% (10/43) of single SSM-negative patients with definite TB (Figure 2D). This increases the sensitivity from 61.3% for a single SSM to 70.3% when FujiLAM and a single SSM are used in combination (Figure 2, C and D). Even the more sensitive EclLAM fails to detect 16.2% (11/68) of single SSM-positive patients but would have detected 39.5% (17/43) of single SSM-negative patients with TB (Figure 2D).

FujiLAM also fails to detect 39% (32/82) sputum Xpert-positive patients when using one Xpert as the basis but at the same time it detects 31.0% (9/29) of Xpert-negative patients with definite TB (Figure 2D).

## Discussion

In this multicenter cohort study of 372 HIV-negative outpatients with respiratory symptoms suggestive of pulmonary TB from high-burden TB settings in Peru and South Africa, the FujiLAM POCT was 98.9% specific and identified 53.2% of positive TB cases, representing a 5-fold increase in sensitivity among HIV-negative patients compared with AlereLAM. FujiLAM was designed as a rule-in TB diagnostic test to allow rapid treatment initiation and reached a PPV of 95.2%. Together with its high sensitivity for TB diagnosis in people living with HIV (19) (sensitivity of 70.7% across CD4 strata), the FujiLAM might have considerable impact on the TB epidemic when scaled-up widely for use in near-patient settings. This is supported by a recent impact modeling analysis. The analysis focusing on LAM-based assays concluded that, relative to the status quo, using a urine-based LAM assay (with 70% sensitivity in people living with HIV and 30% sensitivity in HIV-negative people) in all people presenting to care with TB symptoms would avert 30% of TB deaths and 18% of incident TB cases between 2020 and 2035 in South Africa (22). The FujiLAM does meet these targets in the study analyzed here. While the study might overestimate sensitivity because of the high burden of disease at the study sites, it might at the same time underestimate sensitivity as the study did not consider patients with extrapulmonary TB or patients that have a hard time producing a sputum (e.g., children). FujiLAM’s NPV is 83.2% and a negative FujiLAM result alone should not be used to rule-out TB; additional microbiological testing is required.

When considering LAM assays for real-world clinical use it is important to evaluate the diagnostic yield, PPV, and NPV of algorithms that combine LAM assays and sputum-based assays such as Xpert or SSM (23). In this study, the combination of FujiLAM and Xpert reached 82.0% sensitivity at 98.9% specificity (Figure 2), a PPV of 96.8%, and NPV of 92.8% (Table 2). Importantly, the combination of FujiLAM and SSM, which is still widely used in clinical practice if Xpert or other molecular tests are not available, reached a similarly high PPV of 96.3% and NPV of 88.7%. The use of these combinations has the potential to rapidly inform TB treatment within a day or less in decentralized settings, and treatment in FujiLAM-positive patients can immediately be started due to the tests’ high PPV. The characteristics of FujiLAM and Xpert are complementary: FujiLAM cannot detect drug resistance but Xpert can; Xpert is instrument-based but FujiLAM is a fully disposable POCT; and Xpert uses sputum that is often hard to obtain whereas FujiLAM uses urine. These findings, as well as the outcomes from the modeling studies (22, 24) suggest that there may be value in integrating LAM-based assays such as FujiLAM into diagnostic algorithms in general populations. A recent assessment further concludes that the Xpert/FujiLAM combination can be cost effective (our unpublished observations).

An algorithm that starts with x-ray in combination with symptom-based screening to rule out TB and increase pretest probability followed by FujiLAM-based diagnosis warrants further investigation. In sum, future studies should carefully assess FujiLAM’s added value in real-world scenarios in combination with different tests available at various levels of care and report the PPV and NPV of such algorithms.

When comparing the 2 study sites, the performance of all LAM tests was lower in South Africa compared with Peru. The FujiLAM PPV in South Africa was 72.7% compared with 100% in Peru, which was partially due to the lower TB prevalence in South Africa. Assuming a similar pretest probability (prevalence) like in Peru, which could be achieved with optimized TB screening (e.g., with x-ray), the PPV of FujiLAM would increase to greater than 90%. This is sufficiently high to initiate treatment and substantially higher than the clinical diagnosis that is often used in today’s clinical practice to initiate empiric treatment. Various indicators suggest that late medical consultation resulting in more advanced disease in Peru, or patient selection bias, are possible reasons that explain the large differences between sites. This is further supported by a relatively high TB prevalence, high SSM positivity rate, and more patients with higher mycobacterial loads in sputum, as indicated by 62% of patients with short MGIT time to detection (TTD) in Peru compared with patients in South Africa (Table 1). Subgroup analyses showed that LAM positivity is associated with surrogate markers of body mycobacterial load, such as shorter MGIT TTD, SSM positivity and Xpert semiquantitative result (Figure 4). This finding is in line with an earlier study showing that urine LAM likely reflects total mycobacterial body burden (25). On the other hand, FujiLAM was negative in a subset of patients with smear-positive disease, suggesting that mycobacterial burden in the sputum is likely not fully reflective of total mycobacterial body burden. Another factor that could impact diagnostic accuracy as a function of geography are structural differences of LAM in different TB strains but there is no scientific evidence of such differences, and further research is needed.

A high specificity (≥98%) of a POCT is necessary to avoid overtreatment. Specificity of FujiLAM in this study with HIV-negative patients was 98.9%, higher than AlereLAM’s specificity at 92.3% (Figure 2). Earlier studies reported a lower specificity for FujiLAM (16, 19, 26) and the result from this study underlines the importance of a very comprehensive reference standard for a proper specificity assessment of urine biomarker tests (27).

Our study further shows the potential of LAM as a TB diagnostic biomarker. Using preconcentration of urine samples and the ultrasensitive EclLAM assay, which exploits high-affinity monoclonal antibodies directed toward *Mtb*-specific lipoarabinomannan epitopes (14), we demonstrated that sensitivity increments compared with FujiLAM are feasible. However, this currently requires specialist laboratory equipment allowing electrochemiluminescent-based detection and sophisticated assay protocols. The EclLAM assay reached 66.7% sensitivity at 98.1% specificity, showing that a threshold around 5 pg/mL LAM or below is required to meet the TPP sensitivity target. Other recent research studies (25, 28–30) indicated that lower detection limits will translate into higher diagnostic sensitivity. We also showed this in our earlier small case control study that uses an earlier version of the EclLAM and reached 80% sensitivity in HIV-negative SSM-positive patients at a threshold of 11 pg/mL (14). In this study, despite the lower threshold due to urine preconcentration, the sensitivity of EclLAM is lower, which is likely a result of the case control design of the earlier study, whereas this study used a rigorous cohort design with a low risk of bias.

It is important to mention that our threshold is an estimate based on the EclLAM assay and nonstandardized LAM calibration material and might not be generalizable to other LAM assays with different antibodies, detection technologies, or LAM calibration material. Establishing biological reference materials, as has been done by the WHO for other diseases (31), is an urgent priority to support the development, validation, and comparison of current and future LAM assays.

EclLAM is a research assay employing laboratory equipment and is not designed for use at the POC. In addition, preconcentration was required to increase sensitivity. Therefore, key challenges in the development of next generation LAM POCT’s are to reach a high analytical sensitivity with thresholds in the low pg/mL range while keeping the test simple, affordable, and highly specific. Today, the most sensitive POC lateral flow immunoassays detect antigens, like the Malaria histidine-rich protein II or LAM in case of FujiLAM, in the low picogram per milliliter range (16, 32) and sample concentration, signal amplification, and/or reagent optimization will likely be needed for POCT’s to reach sensitivities like the EclLAM.

Taken together, these results suggest that LAM is present in the urine of most HIV-negative patients and that improved assay methods and reagents for LAM detection will lead to increased diagnostic accuracy. The results also suggest that as the detection limits for high sensitivity laboratory-based tests for LAM continue to improve, centralized urine or blood-based (33) TB antigen detection could also provide a high-throughput complement to nucleic acid tests for TB.

The strengths of this study were the consecutive enrollment of a cohort of HIV-negative patients from 2 epidemiologically diverse TB endemic settings in Africa and South America, the comparison of 3 independent LAM assays, the rigorous study design and the comprehensive reference standard. A limitation of the study is that patients unable to provide sputum and patients in whom the disease was thought to be only extrapulmonary and who might benefit from non–sputum-based testing (20) were excluded, which could have decreased the sensitivity of the LAM assays. Also, the SSM proportion, Xpert, and MGIT TTD results suggest more advanced disease in the Peruvian cohort but relatively low burden in South Africa, which could have artificially influenced the sensitivity of the assays. FujiLAM was designed as a POCT and can be used with fresh, unprocessed urine. The use of frozen urine samples for LAM testing in this study could have lowered LAM concentrations, as a recent study showed that the use of fresh samples leads to minor sensitivity increases in FujiLAM (34). Centrifugation of urine is not necessary before FujiLAM testing, but it was a standard procedure in this study.

In conclusion, FujiLAM has the potential to improve rapid diagnosis of TB at the point-of-care among all people with presumptive TB presenting to outpatient health care centers and could have a high impact on patient outcomes if implemented as a rule-in test in combination with rapid treatment initiation. Further prospective studies are needed to confirm these findings and assess the effect on patient impact to inform policy. Furthermore, the findings highlight the clinical potential of LAM-based diagnosis, and research toward an even more sensitive generation of LAM tests should be prioritized.

## Methods

### Study design and participants.

In this multicenter diagnostic accuracy study, we consecutively enrolled adults aged 18 or older with symptoms of pulmonary TB (at least 2 weeks of persistent cough and at least one additional finding such as hemoptysis, weight loss, fever, night sweats, malaise, contact with an active case, chest pain, or loss of appetite) able to produce sputum. In South Africa, outpatients were enrolled at the Town Two and Nolungile primary health care facilities in the Khayelitsha township between February 9, 2017, and August 31, 2017. In Peru, outpatients were enrolled in 28 primary health care DOTS (directly observed treatment, short-course) treatment centers in high TB prevalence areas in the suburbs of Lima, and referred to the Universidad Peruana Cayetano Heredia between March 22, 2017, and October 4, 2017. Patients in whom the disease was thought to be only extrapulmonary or who received anti-TB treatment in the 60 days before enrollment were excluded (Supplemental Table 1).

### Procedures.

Three sputum (2 on day 1 and a third sputum on day 2), 1 blood, and 2 urine specimens were collected at enrollment and one sputum specimen was collected at 2 months follow-up for reference standard testing. Details on the specimen collection and testing flow are provided in Supplemental Figure 1. Reference standard testing was performed in the reference laboratories of the University of Cape Town and the Universidad Peruana Cayetano Heredia on all sputum specimens and included Xpert (Cepheid; Xpert testing predated rollout of Xpert Ultra), smear fluorescence microscopy after Auramine O staining, MGIT liquid culture (Becton Dickinson), and solid culture on Löwenstein-Jensen medium. Blood cultures from all participants were done in BACTEC Myco/F Lytic culture vials (Becton Dickinson). On average 9.4 valid liquid or solid sputum cultures and 2.7 sputum Xpert results were available per patient, 74% of patients had a valid blood culture and 97% had a valid urine Xpert result. The presence of *Mtb* complex in solid and liquid culture was confirmed with MPT64 antigen (Tauns) detection and/or the MTBDRplus line probe assays (Hain Lifesciences). WHO prequalified in vitro diagnostics were used for HIV testing (rapid diagnostic tests) and CD4 cell counting (flow cytometry). Urine was immediately put on ice after collection and processed within 4 hours. Urine was centrifuged (2000*g* at 4°C for 10 minutes), aliquoted on the day of collection, and stored at –80°C until batch testing of the liquid fraction with LAM assays. For urinary Xpert testing, 30–40 mL urine was centrifuged (3000*g* at 4°C for 15 minutes), and following removal of supernatant the pellet was resuspended in 1 mL PBS and tested using Xpert on the day of collection. Clinical information, index test results, and comparator test results were not available to the assessors of the reference standard.

Upon completion of the enrollment, frozen urine aliquots of the complete cohort were shipped to the Research Institute of Tuberculosis of the Japan Anti-Tuberculosis Association (RIT-JATA, Tokyo, Japan) for AlereLAM (Abbott) and FujiLAM (FujiFilm) testing between January 29, 2019, and February 14, 2019, and to Meso Scale Diagnostics LLC (Rockville, Maryland, USA) for EclLAM testing between September 18, 2018, and September 28, 2018.

For AlereLAM and FujiLAM testing at RIT-JATA, frozen urine aliquots were thawed to ambient temperature and mixed by inversion. Samples that were not immediately used for testing were stored at 4°C for a maximum of 4 hours. Testing with FujiLAM was performed according to the manufacturers’ instructions using urine from the same aliquot as that used for AlereLAM. The 5-step FujiLAM test procedure is illustrated in an online video (35) and takes 50–60 minutes from start to end result. The FujiLAM assay does not use a reference scale card and any visible test line is considered positive. The AlereLAM test was used according to the tests package insert and the four-grade Reference Scale Card, with the Grade 1 cutoff point as the positivity threshold. FujiLAM and AlereLAM were independently read by 2 readers, each blinded to all other clinical, demographic, and test data associated with the samples. After the initial test interpretation, the 2 readers compared results and, in the event of discordance, established a final consensus result by mutual agreement. In case of test failure, the test was repeated, and the first valid result was used for the analysis.

Blinded EclLAM testing at Meso Scale Diagnostics followed a previously established assay protocol, except for the addition of a preconcentration step to improve the limit of detection (14). To preconcentrate, frozen urine aliquots were thawed to ambient temperature, mixed by inversion, and a 490 μL sample was added to Amicon Ultra-0.5 mL centrifugal filters (MilliporeSigma) with a 3 kDa cutoff. After ultrafiltration for 20 minutes at 14000*g*, approximately 25 μL deionized water was added to the retentate (~45 μL) to get a total of 70 μL and an estimated concentration factor of 7. Prior to analysis, samples were heat treated at 85°C for 10 minutes. The immunoassays for LAM used the same antibodies as FujiLAM in a sandwich immunoassay format employing ECL detection as described in the Supplemental Methods and elsewhere (14, 17, 36). The research team performing EclLAM had no access to all other clinical, demographic, and test data associated with the samples.

### Statistics.

Before data analysis, clinical investigators, who were masked to index test results, categorized patients as having definite TB, possible TB, not TB, and unclassifiable, using a combination of clinical and laboratory findings (Supplemental Table 2). Patients with definite TB had microbiologically confirmed *Mtb* (any culture or any Xpert positive for *Mtb* during admission). Patients defined as not TB had all microscopy, culture, and Xpert test results negative for *Mtb*, had not started TB treatment, and were alive or had improvement in clinical tuberculosis symptoms at a 2-month follow-up. Patients defined as possible TB did not satisfy the criteria for definite TB but had clinical features suggestive of TB and were started on TB treatment. Patients who did not fall into any of these categories were defined as unclassifiable and were removed from the main analyses. Definite TB and not TB categories were used to allocate patients into positive and negative, respectively, for both the MRS and CRS. The possible TB group was considered negative by MRS, but positive by CRS as previously proposed in a study guidance publication (27).

Simple descriptive statistics were used to characterize cohorts. We calculated the point estimates and 95% Wilson CIs for the sensitivity, specificity, PPV, NPV, LR+, and LR− for FujiLAM, AlereLAM, EclLAM, Sputum Smear Microscopy (SSM), Xpert, and combinations of FujiLAM+SSM and FujiLAM+Xpert by comparison with the MRS and CRS. The R package eulerr (37) was used to generate area-proportional Euler diagrams to illustrate the number of positive test results by test in the definite TB group. Fagan nomograms were used to illustrate pretest and posttest probabilities of FujiLAM and AlereLAM.

The cutoff of the EclLAM assay was set at 5.2 pg/mL after review of the data to achieve a specificity of at least 98%. Diagnostic accuracy for LAM assays was determined separately for each cohort and analyzed post hoc by MGIT time to detection (MGIT TTD), SSM status, and semiquantitative Xpert result. EclLAM concentration-based ROC curves were used to show the relationship of LAM concentration, sensitivity, and specificity.

### Study approval.

The study was approved by the Human Research Ethics Committee of the University of Cape Town (Cape Town, South Africa), the City of Cape Town (Cape Town, South Africa; ref. 10364a), the Universidad Peruana Cayetano Heredia (Lima, Peru), and the Peruvian Ministry of Health (Lima, Peru; ref. 18829-2016). Written informed consent was obtained from patients, as per study protocols. Study participation did not affect standard of care. This study is reported in accordance with the Standards for Reporting of Diagnostic Accuracy Studies (STARD) guidelines. The study protocol is available on request and patient level data are available online in the Supplemental Material.

## Author contributions

TB, EIR, SGS, and CMD designed and oversaw the study. MPN, EG, SS, JVH, and TCN coordinated the individual study sites in South Africa and Peru. TB, GBS, MT, TP, TLL, AP, and EM contributed to EclLAM assay and reagent development. KC, RS, and SM coordinated measurement of AlereLAM and FujiLAM. AM and RS coordinated data collection and management. TB did the statistical analysis and TB and AJZ wrote the first manuscript draft. All authors contributed to interpretation of data and editing of the article and approved the final version of the manuscript. Authorship, including the order of co–first authors, was based on International Committee of Medical Journal Editors criteria.

## Supplementary Material

Supplemental data

ICMJE disclosure forms

Supplemental Data Set 1

## Figures and Tables

**Table 2:**
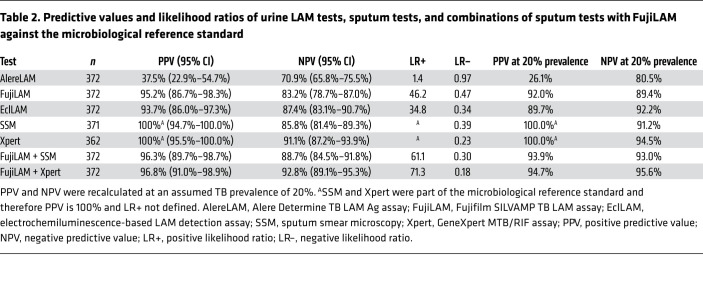
Predictive values and likelihood ratios of urine LAM tests, sputum tests, and combinations of sputum tests with FujiLAM against the microbiological reference standard

**Table 1:**
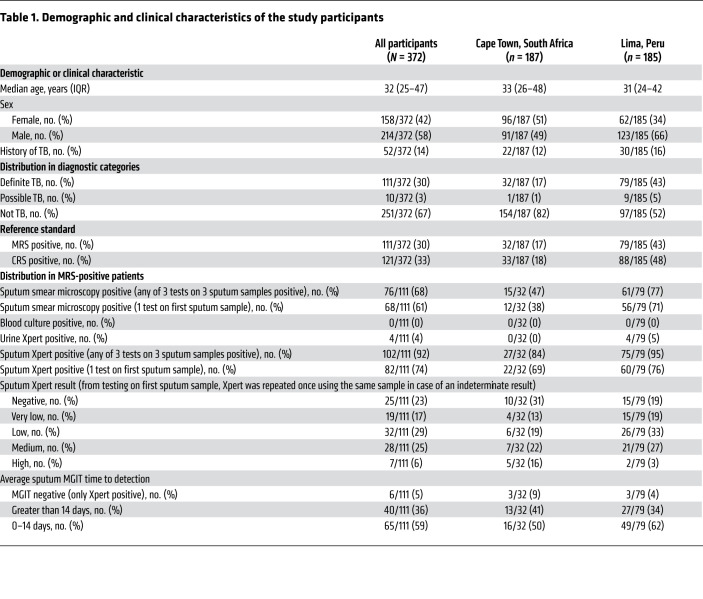
Demographic and clinical characteristics of the study participants

## Version 1 07/21/2020

In-Press Preview

## Version 2 09/28/2020

Electronic publication

## Version 3 11/02/2020

Print issue publication
