# Supplementary material for: Diagnostic accuracy of 3 urine lipoarabinomannan tuberculosis assays in HIV-negative outpatients
Source: J Clin Invest. 2020 Sep 28;130(11):5756–64. doi: 10.1172/JCI140461 (PMC7598043; doi:10.1172/JCI140461)
Supplement: Supplemental data [file jci-130-140461-s020.pdf]

Supplementary Appendix for:

Diagnostic accuracy of three urine lipoarabinomannan tuberculosis assays in HIV-negative outpatients

Table of Contents

Study population, setting & location, eligibility, inclusion & exclusion criteria 2

Specimen Collection and Testing Flow 3

Diagnostic Categories 4

EclLAM assay methods 5

EclLAM calibration curve 6

Euler diagram showing the proportions of TB patients detected with urine LAM tests 7

Detailed diagnostic accuracy against the microbiological reference standard (MRS)........ 8

Detailed diagnostic accuracy against the composite reference standard (CRS) 8

Fagan nomograms for AlereLAM and FujiLAM 9

Subgroup analysis for AlereLAM 10

Subgroup analysis for EclLAM 11

Supplementary References........ 12

Supplemental Table 1. Study population, setting & location, eligibility, inclusion & exclusion criteria

Cohort	South Africa – Cape Town	Peru – Lima
Study population	Adults suspected of having active TB disease<br>High HIV prevalence setting	Adults suspected of having active TB disease<br>Low HIV prevalence setting
Setting & location	South Africa, Cape Town<br>-Town Two Clinic<br>-Nolungile Clinic<br>-Outpatients	Peru, Lima<br>-28 primary health care DOTS treatment centres<br>-Outpatients
Eligibility criteria	Adults (≥18 years) presenting with symptomatic pulmonary disease thought to have TB	
Inclusion criteria	-Informed consent from patient<br>-Suspected to have active TB based on clinical presentation<br>-Production of adequate quantity of sputum	
Exclusion criteria	-Participants receiving any anti-tuberculosis medication in the 60 days prior to enrolment<br>-Participants with only extra-pulmonary disease	
Enrolment period	February 2017 – August 2017	March 2017– Oct 2017
Participants considered for urinary LAM testing	HIV-negative patients of the prospective cohort study. HIV-positive patients were excluded from the main analysis.	HIV-negative patients of the prospective cohort study. HIV-positive patients were excluded from the main analysis.
Study protocol, Principle Investigator	Protocol: FIND Reference Materials Collection Protocol Version 14.0.<br><br>Principle Investigator Prof. Dr. Mark Nicol.	Protocol: LAM ad-hoc Collection Protocol date 01 September 2016<br><br>Principle Investigator Dr. Eduardo Gotuzzo.

Supplemental Figure 1. Specimen Collection and Testing Flow

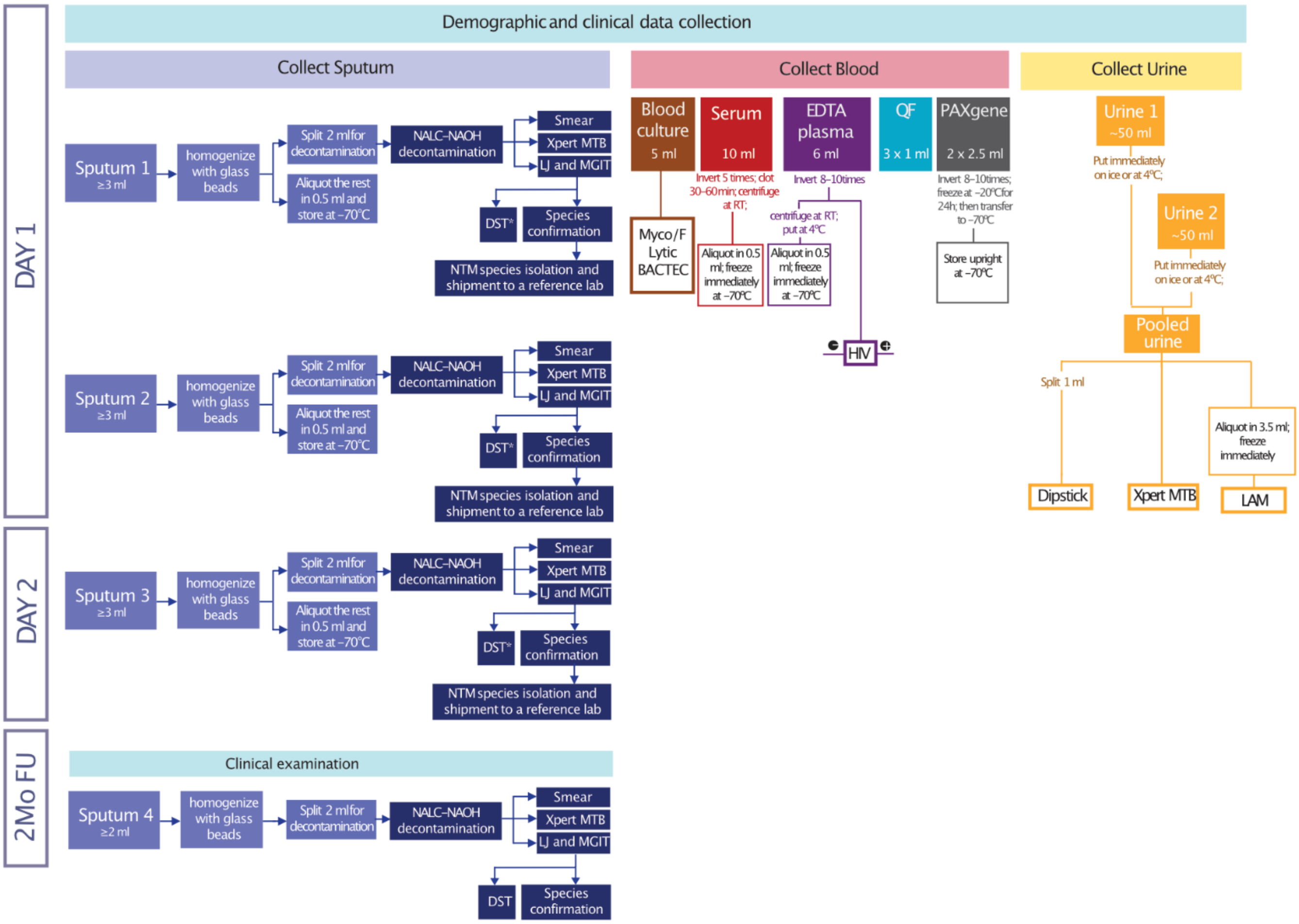

Supplemental Table 2. Diagnostic Categories

Category	Definition
**Definite TB**	**Any culture or any Xpert (baseline) positive for MTB**<br>≥1 Positive culture (solid, liquid, sputum or blood) & confirmed MTB complex at baseline (Cross-contamination: A single LJ culture with ≤ 20 colonies or a single MGIT culture with MTB growth ≥28 days per patient are excluded from analysis)<br>OR<br>≥1 Positive Xpert (sputum or urine) at baseline
**Possible TB**	**Any patient not meeting definite TB or Not TB classification who is started on TB treatment or has positive laboratory findings on follow-up**<br>Empiric TB treatment started by the healthcare provider<br>Or<br>Positive sputum culture and/or sputum Xpert and/or sputum smear on follow-up
**Not TB**	**All microscopy, culture and Xpert tests negative for MTB, not started on TB treatment, recovers and has negative follow-up tests**<br>All culture negative (sputum, blood, incl. follow-up, with at least 2 LJ or MGIT with no culture growth after >56 days and >42 days)<br>AND<br>At least 2 valid negative culture or Xpert results from 2 or more independent samples obtained from 2 or more different anatomic sites, such as blood, sputum, or urine<br>AND<br>All Xpert negative (incl. follow-up)<br>AND<br>All smear microscopy results negative (incl. follow-up)<br>AND<br>Treatment not initiated by healthcare providers<br>AND<br>Improvement or full recovery at 8-week follow-up in the absence of TB treatment
**Unclassifiable**	**All participants that do not fall into groups "Definite TB", "Not TB" or "Possible TB"**<br>i.e.:<br>No symptom resolution on follow-up (same or worse) for baseline negative participants<br>Or<br>Loss to follow-up for baseline negative participants<br>Or<br>Passed away for baseline negative participants<br>Or<br>Insufficient laboratory results (i.e. participants with <2 valid cultures)<br>OR<br>Baseline smear microscopy positive but culture and Xpert negative

Supplemental Methods 1. EclLAM assay methods

Prior to analysis, urine samples were concentrated and heat-treated as described in the Methods section in the main body of this paper. The processed samples were analyzed using immunoassays for LAM employing a sandwich immunoassay format and electrochemiluminescence (ECL) detection. The assays were carried out on commercial instrumentation and U-PLEX® multi-well plate consumables from Meso Scale Diagnostics, LLC. (MSD). Each well of the U-PLEX plate has an array with 10 array elements (or "spots"), each comprising a binding reagent designed to bind one of a set of 10 proprietary linkers (available from MSD). The S4-20 capture antibody (kindly provided by Dr. Masanori Kawasaki, Otsuka Pharmaceutical, Tokyo, Japan, described in (1)) was coupled to a U-PLEX linker and immobilized on one spot of the array in each well following the immobilization procedure in the U-PLEX package insert. Assays were run according to the following procedure using commercial diluents from MSD. A 25 μL volume of MSD Diluent 22 was combined with 25 μL of sample in each well of the U-PLEX plate, and the mixture was incubated with shaking for 1 h at room temperature to bind LAM in the sample to the capture antibody array in the well. After washing the wells to remove the unbound sample, 25 μL of 2-μg/ml SULFO-TAG-labeled A194-01 detection antibody (kindly provided by, Dr. Abraham Pinter, Rutgers University, Newark, USA, described in (2)) in MSD Diluent 3 supplemented with casein was added and incubated for an additional 1 h with shaking to complete the immunoassay sandwich. After washing the wells to remove the unbound detection antibody, the wells were filled with 150 μL of 2X MSD Read Buffer T, and ECL was measured on an MSD Sector® S 600 ECL plate reader. The plate reader applies a voltage to the electrodes in the MSD plates to induce ECL from the bound detection antibodies and quantitates the resulting ECL emission. To calculate LAM concentrations, an eight-point calibration curve with purified *Mtb* LAM (obtained from Nacalai USA Inc., San Diego, USA) diluted in phosphate-buffered saline plus 2% bovine serum albumin was run in duplicates in each assay plate. The relationship of ECL signal to LAM concentration was fitted to a four-parameter logistic (4-PL) function, after first dividing the assigned LAM concentrations by a concentration factor of 7 to account for 7-fold concentration of test samples prior to analysis. LAM concentrations for test samples were calculated by back-fitting ECL signals to the 4-PL fit. The limit of detection was calculated as the concentration of LAM (as determined from the 4-PL fit) that provides an assay signal that is 2.5 standard deviations above the signal for the calibrator diluent.

Supplemental Figure 2. EclLAM calibration curve

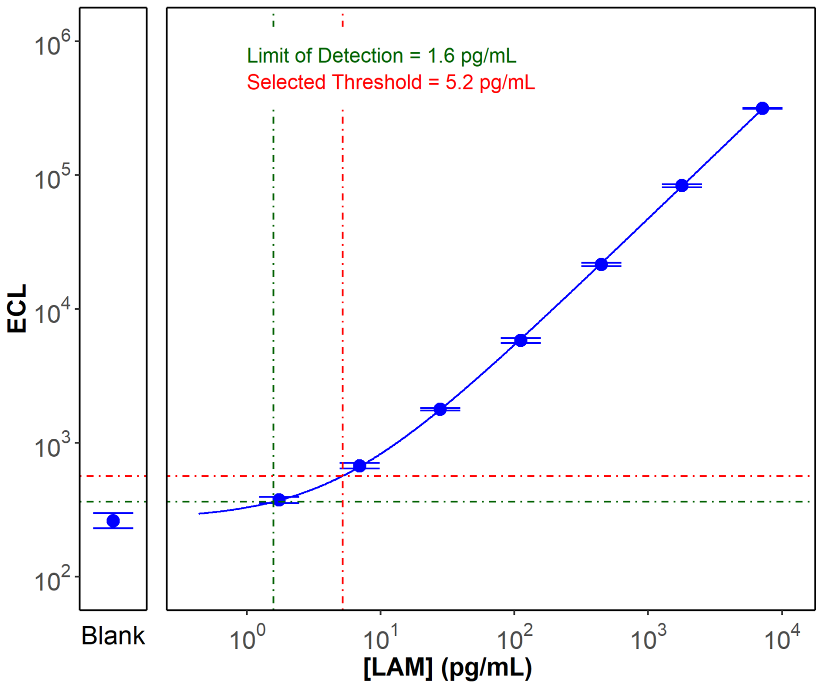

Supplemental Figure 3. Euler diagram showing the proportions of TB patients detected with urine LAM tests

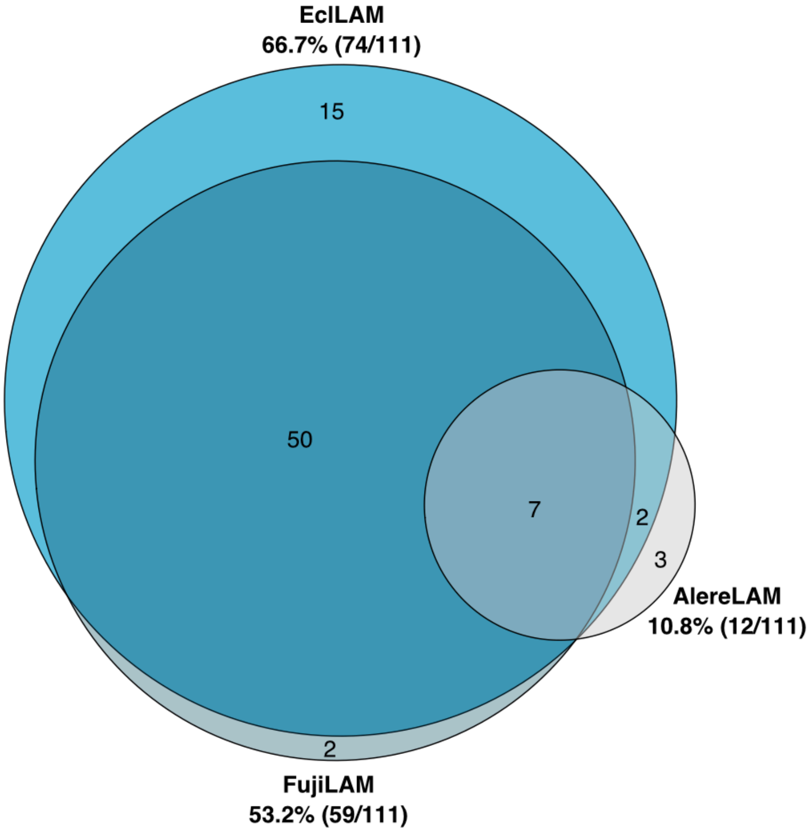

Supplemental Table 2. Detailed diagnostic accuracy against the microbiological reference standard (MRS)

Test	n	TP	FP	FN	TN	Sensitivity (95% CI)		Specificity (95% CI)		PPV (95% CI)		NPV (95% CI)		LR+	LR −	PPV at 20%Prev.	NPV at 20%Prev.
AlereLAM	372	12	20	99	241	10.8%	(6.3 to 18.0)	92.3%	(88.5 to 95.0)	37.5%	(22.9 to 54.7)	70.9%	(65.8 to 75.5)	1.4	0.97	26.1%	80.5%
FujiLAM	372	59	3	52	258	53.2%	(43.9 to 62.2)	98.9%	(96.7 to 99.6)	95.2%	(86.7 to 98.3)	83.2%	(78.7 to 87.0)	46.2	0.47	92.0%	89.4%
EclLAM	372	74	5	37	256	66.7%	(57.5 to 74.7)	98.1%	(95.6 to 99.2)	93.7%	(86.0 to 97.3)	87.4%	(83.1 to 90.7)	34.8	0.34	89.7%	92.2%
SSM	371	68	0	43	260	61.3%	(52.0 to 69.8)	100%*	(98.5 to 100.0)	100%*	(94.7 to 100.0)	85.8%	(81.4 to 89.3)	NA*	0.39	100%*	91.2%
FujiLAM + SSM	372	78	3	33	258	70.3%	(61.2 to 78.0)	98.9%	(96.7 to 99.6)	96.3%	(89.7 to 98.7)	88.7%	(84.5 to 91.8)	61.1	0.30	93.9%	93.0%
Xpert	362	82	0	25	255	76.6%	(67.8 to 83.6)	100%*	(98.5 to 100.0)	100%*	(95.5 to 100.0)	91.1%	(87.2 to 93.9)	NA*	0.23	100%*	94.5%
FujiLAM + Xpert	372	91	3	20	258	82.0%	(73.8 to 88.0)	98.9%	(96.7 to 99.6)	96.8%	(91.0 to 98.9)	92.8%	(89.1 to 95.3)	71.3	0.18	94.7%	95.6%

Comment: *SSM and Xpert are part of the reference standard leading to a specificity and PPV of 100% and LR+ is not defined

Supplemental Table 3. Detailed diagnostic accuracy against the composite reference standard (CRS)

Test	n	TP	FP	FN	TN	Sensitivity (95% CI)		Specificity (95% CI)		PPV (95% CI)		NPV (95% CI)		LR+	LR −	PPV at 20%Prev.	NPV at 20%Prev.
AlereLAM	372	15	17	106	234	12.4%	(7.7 to 19.4)	93.2%	(89.4 to 95.7)	46.9%	(30.9 to 63.6)	68.8%	(63.7 to 73.5)	1.8	0.94	31.4%	81.0%
FujiLAM	372	59	3	62	248	48.8%	(40.0 to 57.6)	98.8%	(96.5 to 99.6)	95.2%	(86.7 to 98.3)	80.0%	(75.2 to 84.1)	40.8	0.52	91.1%	88.5%
EclLAM	372	75	4	46	247	62.0%	(53.1 to 70.1)	98.4%	(96.0 to 99.4)	94.9%	(87.7 to 98.0)	84.3%	(79.7 to 88.0)	38.9	0.39	90.7%	91.2%
SSM	371	68	0	53	250	56.2%	(47.3 to 64.7)	100%*	(98.5 to 100.0)	100%*	(94.7 to 100.0)	82.5%	(77.8 to 86.4)	NA*	0.44	100%*	90.1%
FujiLAM + SSM	372	78	3	43	248	64.5%	(55.6 to 72.4)	98.8%	(96.5 to 99.6)	96.3%	(89.7 to 98.7)	85.2%	(80.7 to 88.8)	53.9	0.36	93.1%	91.8%
Xpert	362	82	0	35	245	70.1%	(61.3 to 77.6)	100%*	(98.5 to 100.0)	100%*	(95.5 to 100.0)	87.5%	(83.1 to 90.9)	NA*	0.30	100%*	93.0%
FujiLAM + Xpert	372	91	3	30	248	75.2%	(66.8 to 82.0)	98.8%	(96.5 to 99.6)	96.8%	(91.0 to 98.9)	89.2%	(85.0 to 92.3)	62.9	0.25	94.0%	94.1%

Comment: *SSM and Xpert are part of the reference standard leading to a specificity and PPV of 100% and LR+ is not defined

Supplemental Figure 4. Fagan nomograms for AlereLAM and FujiLAM

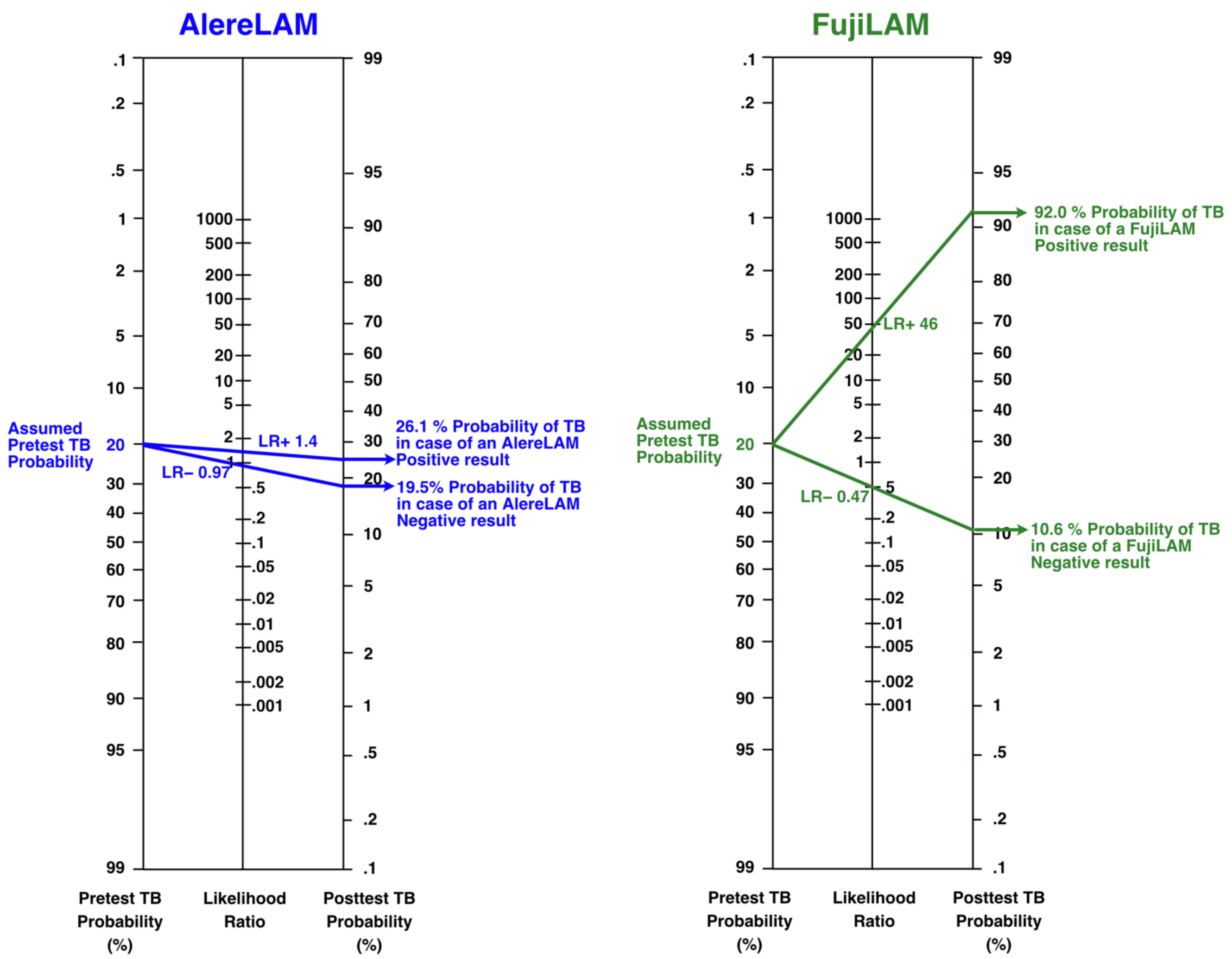

Supplemental Figure 5. Subgroup analysis for AlereLAM

A

Cohort	n	TP	FP	FN	TN	Sensitivity (95% CI)	Specificity (95% CI)
South Africa	187	2	13	30	142	6.3% (1.7 to 20.1)	91.6% (86.2 to 95.0)
Peru	185	10	7	69	99	12.7% (7.0 to 21.8)	93.4% (87.0 to 96.8)

B

MGIT TTD	n	TP	FP	FN	TN	Sensitivity (95% CI)
>14 days	40	5	NA	35	NA	12.5% (5.5 to 26.1)
0-14 days	65	7	NA	58	NA	10.8% (5.3 to 20.6)

C

SSM status (any of three SSM positive)	n	TP	FP	FN	TN	Sensitivity (95% CI)
Negative	35	1	NA	34	NA	2.9% (0.5 to 14.5)
Positive	76	11	NA	65	NA	14.5% (8.3 to 24.1)

D

Xpert semiquantitative result	n	TP	FP	FN	TN	Sensitivity (95% CI)
Negative	25	1	NA	24	NA	4.0% (0.7 to 19.5)
Very low	19	2	NA	17	NA	10.5% (2.9 to 31.4)
Low	32	2	NA	30	NA	6.3% (1.7 to 20.1)
Medium	28	5	NA	23	NA	17.9% (7.9 to 35.6)
High	7	2	NA	5	NA	28.6% (8.2 to 64.1)

Supplemental Figure 6. Subgroup analysis for EclLAM

A

Cohort	n	TP	FP	FN	TN	Sensitivity (95% CI)	Specificity (95% CI)
South Africa	187	12	0	20	155	37.5% (22.9 to 54.7)	100.0% (97.6 to 100.0)
Peru	185	62	5	17	101	78.5% (68.2 to 86.1)	95.3% (89.4 to 98.0)

B

MGIT TTD	n	TP	FP	FN	TN	Sensitivity (95% CI)
>14 days	40	22	0	18	0	55.0% (39.8 to 69.3)
0-14 days	65	51	0	14	0	78.5% (67.0 to 86.7)

C

SSM status (any of three SSM positive)	n	TP	FP	FN	TN	Sensitivity (95% CI)
Negative	35	11	0	24	0	31.4% (18.6 to 48.0)
Positive	76	63	0	13	0	82.9% (72.9 to 89.7)

D

Xpert semiquantitative result	n	TP	FP	FN	TN	Sensitivity (95% CI)
Negative	25	9	0	16	0	36.0% (20.2 to 55.5)
Very low	19	12	0	7	0	63.2% (41.0 to 80.9)
Low	32	22	0	10	0	68.8% (51.4 to 82.0)
Medium	28	25	0	3	0	89.3% (72.8 to 96.3)
High	7	6	0	1	0	85.7% (48.7 to 97.4)

Supplemental References

1. Kawasaki M et al. Lipoarabinomannan in sputum to detect bacterial load and treatment response in patients with pulmonary tuberculosis: Analytic validation and evaluation in two cohorts [Internet]. *PLOS Med.* 2019;16(4):e1002780.

2. Choudhary A et al. Characterization of the Antigenic Heterogeneity of Lipoarabinomannan, the Major Surface Glycolipid of Mycobacterium tuberculosis , and Complexity of Antibody Specificities toward This Antigen [Internet]. *J. Immunol.* 2018;200(9):3053–3066.

Section & Topic	No	Item	Reported on page #
TITLE OR ABSTRACT			
	1	Identification as a study of diagnostic accuracy using at least one measure of accuracy (such as sensitivity, specificity, predictive values, or AUC)	√
ABSTRACT			
	2	Structured summary of study design, methods, results, and conclusions (for specific guidance, see STARD for Abstracts)	√
INTRODUCTION			
	3	Scientific and clinical background, including the intended use and clinical role of the index test	√
	4	Study objectives and hypotheses	√
METHODS			
Study design	5	Whether data collection was planned before the index test and reference standard were performed (prospective study) or after (retrospective study)	√
Participants	6	Eligibility criteria	√
	7	On what basis potentially eligible participants were identified (such as symptoms, results from previous tests, inclusion in registry)	√
	8	Where and when potentially eligible participants were identified (setting, location and dates)	√
	9	Whether participants formed a consecutive, random or convenience series	√
Test methods	10a	Index test, in sufficient detail to allow replication	√
	10b	Reference standard, in sufficient detail to allow replication	√
	11	Rationale for choosing the reference standard (if alternatives exist)	√
	12a	Definition of and rationale for test positivity cut-offs or result categories of the index test, distinguishing pre-specified from exploratory	√
	12b	Definition of and rationale for test positivity cut-offs or result categories of the reference standard, distinguishing pre-specified from exploratory	√
	13a	Whether clinical information and reference standard results were available to the performers/readers of the index test	√
	13b	Whether clinical information and index test results were available to the assessors of the reference standard	√
Analysis	14	Methods for estimating or comparing measures of diagnostic accuracy	√
	15	How indeterminate index test or reference standard results were handled	√
	16	How missing data on the index test and reference standard were handled	√
	17	Any analyses of variability in diagnostic accuracy, distinguishing pre-specified from exploratory	√
	18	Intended sample size and how it was determined	X
RESULTS			
Participants	19	Flow of participants, using a diagram	√
	20	Baseline demographic and clinical characteristics of participants	√
	21a	Distribution of severity of disease in those with the target condition	√
	21b	Distribution of alternative diagnoses in those without the target condition	X
	22	Time interval and any clinical interventions between index test and reference standard	√
Test results	23	Cross tabulation of the index test results (or their distribution) by the results of the reference standard	√
	24	Estimates of diagnostic accuracy and their precision (such as 95% confidence intervals)	√
	25	Any adverse events from performing the index test or the reference standard	X
DISCUSSION			
	26	Study limitations, including sources of potential bias, statistical uncertainty, and generalisability	√
	27	Implications for practice, including the intended use and clinical role of the index test	√
OTHER INFORMATION			
	28	Registration number and name of registry	Studies were not registered
	29	Where the full study protocol can be accessed	√
	30	Sources of funding and other support; role of funders	√

STARD 2015

AIM

STARD stands for “Standards for Reporting Diagnostic accuracy studies”. This list of items was developed to contribute to the completeness and transparency of reporting of diagnostic accuracy studies. Authors can use the list to write informative study reports. Editors and peer-reviewers can use it to evaluate whether the information has been included in manuscripts submitted for publication.

EXPLANATION

A **diagnostic accuracy study** evaluates the ability of one or more medical tests to correctly classify study participants as having a **target condition.** This can be a disease, a disease stage, response or benefit from therapy, or an event or condition in the future. A medical test can be an imaging procedure, a laboratory test, elements from history and physical examination, a combination of these, or any other method for collecting information about the current health status of a patient.

The test whose accuracy is evaluated is called **index test.** A study can evaluate the accuracy of one or more index tests. Evaluating the ability of a medical test to correctly classify patients is typically done by comparing the distribution of the index test results with those of the **reference standard**. The reference standard is the best available method for establishing the presence or absence of the target condition. An accuracy study can rely on one or more reference standards.

If test results are categorized as either positive or negative, the cross tabulation of the index test results against those of the reference standard can be used to estimate the **sensitivity** of the index test (the proportion of participants *with* the target condition who have a positive index test), and its **specificity** (the proportion *without* the target condition who have a negative index test). From this cross tabulation (sometimes referred to as the contingency or “2x2” table), several other accuracy statistics can be estimated, such as the positive and negative **predictive values** of the test. Confidence intervals around estimates of accuracy can then be calculated to quantify the statistical **precision** of the measurements.

If the index test results can take more than two values, categorization of test results as positive or negative requires a **test positivity cut-off**. When multiple such cut-offs can be defined, authors can report a receiver operating characteristic (ROC) curve which graphically represents the combination of sensitivity and specificity for each possible test positivity cut-off. The **area under the ROC curve** informs in a single numerical value about the overall diagnostic accuracy of the index test.

The **intended use** of a medical test can be diagnosis, screening, staging, monitoring, surveillance, prediction or prognosis. The **clinical role** of a test explains its position relative to existing tests in the clinical pathway. A replacement test, for example, replaces an existing test. A triage test is used before an existing test; an add-on test is used after an existing test.

Besides diagnostic accuracy, several other outcomes and statistics may be relevant in the evaluation of medical tests. Medical tests can also be used to classify patients for purposes other than diagnosis, such as staging or prognosis. The STARD list was not explicitly developed for these other outcomes, statistics, and study types, although most STARD items would still apply.

DEVELOPMENT

This STARD list was released in 2015. The 30 items were identified by an international expert group of methodologists, researchers, and editors. The guiding principle in the development of STARD was to select items that, when reported, would help readers to judge the potential for bias in the study, to appraise the applicability of the study findings and the validity of conclusions and recommendations. The list represents an update of the first version, which was published in 2003.

More information can be found on http://www.equator-network.org/reporting-guidelines/stard.

CC BY SA
